# Gender Disparities and Lung Cancer Screening Outcomes Among Individuals Who Have Never Smoked

**DOI:** 10.1001/jamanetworkopen.2024.54057

**Published:** 2025-01-15

**Authors:** Yeon Wook Kim, Dong-Hyun Joo, So Yeon Kim, Young Sik Park, Sowon Jang, Jong Hyuk Lee, Gerard A. Silvestri, Marjolein A. Heuvelmans, Jihang Kim, Hyeontaek Hwang, Choon-Taek Lee

**Affiliations:** 1 Division of Pulmonary and Critical Care Medicine, Department of Internal Medicine, Seoul National University Bundang Hospital, Seongnam, Republic of Korea; 2 Department of Internal Medicine, Seoul National University College of Medicine; 3 Division of Pulmonary and Critical Care Medicine, Department of Internal Medicine, Seoul National University Hospital, Seoul, Republic of Korea; 4 Department of Radiology, Seoul National University Bundang Hospital, Seongnam, Republic of Korea; 5 Department of Radiology and Institute of Radiation Medicine, Seoul National University Hospital, Seoul, Republic of Korea; 6 Division of Pulmonary Medicine, Thoracic Oncology Research Group, Hollings Cancer Center, Medical University of South Carolina, Charleston, South Carolina; 7 University of Groningen, University Medical Center Groningen, Department of Epidemiology, Groningen, the Netherlands; 8 Institute for Diagnostic Accuracy, Groningen, the Netherlands.; 9 Department of Respiratory Medicine, Amsterdam University Medical Center, Amsterdam, the Netherlands; 10 Seoul National University College of Medicine, Seoul, Republic of Korea

## Abstract

**Importance:**

Lung cancer in individuals who have never smoked (INS) is a growing global concern, with a rapidly increasing incidence and proportion among all lung cancer cases. Particularly in East Asia, opportunistic lung cancer screening (LCS) programs targeting INS have gained popularity. However, the sex-specific outcomes and drawbacks of screening INS remain unexplored, with data predominantly focused on women.

**Objective:**

To compare LCS outcomes between Asian women and men with no smoking history.

**Design, Setting, and Participants:**

This multicenter cohort study was conducted at health checkup centers in South Korea from 2009 to 2021. Participants included individuals aged 50 to 80 years with no smoking history who underwent low-dose computed tomography (LDCT) screening. Data were retrospectively analyzed from November 2023 to June 2024.

**Exposures:**

Opportunistic LDCT screening for lung cancer.

**Main Outcomes and Measures:**

Participants were followed up until December 2022 for the outcome of death. Lung cancer diagnosis, diagnostic characteristics, clinical course, and lung cancer–specific deaths (LCSD) were compared between women and men.

**Results:**

A total of 21 062 participants (16 133 [76.6%] women and 4929 [23.4%] men) with a mean (SD) age of 59.8 (7.2) years were included. From baseline screening, 176 participants (139 women [0.9%] and 37 men [0.8%]) were diagnosed with lung cancer (screen-detected); 131 of 139 women (94.3%) and 33 of 37 men (89.2%) were diagnosed with stage 0 to I disease, with 133 of 139 women (95.7%) and 36 of 37 men (97.3%) having adenocarcinoma. There were no significant sex-based differences in stage or histologic type distribution. Among the 21 062 screened individuals, LCSD was reported in 8 women and 3 men during a mean (SD) follow-up of 83.8 (41.7) months. Multivariable analyses found no significant association between sex and cumulative hazards of lung cancer diagnosis (adjusted hazard ratio [aHR], 0.90 [95% CI, 0.64-1.26] for men vs women) or LCSD (aHR, 1.06 [95% CI, 0.28-4.00] for men vs women). The estimated 5-year lung cancer–specific survival rate was 97.7% for women and 100% for men with screen-detected lung cancer, showing no significant sex differences.

**Conclusions and Relevance:**

In this cohort study of Asian individuals with no smoking history who underwent LDCT screening, no significant sex-based differences were detected in lung cancer diagnosis, stage distribution, or LCSD. These findings suggest that men and women who have never smoked would experience similar risks of overdiagnosis with little to no benefit when exposed to indiscriminate screening.

## Key Points

**Question:**

What are the differences in opportunistic lung cancer screening outcomes, such as exposure to harms of overdiagnosis, between men and women who have never smoked?

**Findings:**

This cohort study of 21 062 individuals in South Korea with no smoking history who underwent opportunistic lung cancer screening found that a substantial proportion of screen-detected lung cancers identified in men exhibited adenocarcinoma as the predominant histological type (97.3%) and were diagnosed at an early stage with excellent survival outcomes (5-year lung cancer–specific survival rate of 100%). These parameters were similar with those observed in women, with no significant sex differences in the risk of lung cancer diagnosis or lung cancer–specific death.

**Meaning:**

These results suggest that men and women with no smoking history experience a similar risk of overdiagnosis with limited benefits when exposed to indiscriminate screening.

## Teaser

This cohort study compares lung cancer screening outcomes, such as exposure to harms of overdiagnosis, between South Korean women and men with no smoking history.

## Introduction

Lung cancer is the leading cause of cancer-related deaths worldwide for men and women.^1^ Survival is strongly associated with disease stage at diagnosis, with stage IA disease having a 5-year survival rate of greater than 75%. Conversely, 5-year survival rate for stage IV disease is less than 10%.^2,3^ Therefore, an important approach to substantially reduce lung cancer mortality over an extended period involves screening asymptomatic individuals using low-dose chest computed tomography (LDCT) for early detection.^4^ Large randomized studies have proven the efficacy of LDCT screening in early detection of lung cancer, thereby reducing lung cancer mortality in populations with substantial cigarette smoke exposure.^5,6,7^ Therefore, both the 2021 US Preventive Services Task Force and the 2023 American Cancer Society guidelines recommend LDCT screening for individuals aged 50 to 80 years with a smoking history of at least 20 pack-years, with or without consideration of smoking cessation duration.^8,9^ Moreover, LDCT-based lung cancer screening (LCS) programs are being developed and getting integrated into various regions and populations globally.^10,11^

Currently, evidence supporting LCS in individuals who have never smoked (INS) is lacking.^12^ Nonetheless, lung cancer in INS (LCINS) is a serious global concern, accounting for approximately one-third of lung cancer deaths.^1^ LCINS incidence and its proportion among all lung cancer cases are rapidly increasing, particularly in Asia.^13,14^ Given its substantial burden, opportunistic LCS programs that routinely include INS have gained popularity in East Asia, including South Korea, Taiwan, China, and Japan.^15,16,17,18^ Although identifying high-risk individuals and offering screening can potentially benefit this population, the indiscriminate use of LDCT screening raises concerns of overdiagnosis.^16,17^ Recent population-based studies from Taiwan, China, and South Korea have explored the potential for lung cancer overdiagnosis in women with no smoking history by assessing changes in epidemiological patterns of diagnosed lung cancer in women following the widespread introduction of opportunistic LDCT screening since the mid-2000s.^16,17,19^ These studies provide valuable epidemiologic data on lung cancer in Asian female INS, considering the low smoking rate among women in the regions (<7%). However, the studies have inherent limitations, such as the inability to directly measure LDCT screening exposure and the smoking history of each individual. Another major limitation of population-based studies is the inability to assess the effect of opportunistic LDCT screening exposure among men with no smoking history, given the high smoking rate in men (>35%).^20^

Globally, women typically engage more often with medical care and undergo opportunistic screening more frequently.^16,21^ This increased exposure to screening likely explains why overdiagnosis of indolent cancers are more prominent in women compared with men.^22,23,24^ If signals of overdiagnosis in lung cancer are associated with increased screening exposure in low-risk populations, it can be assumed that men with no smoking history would also be substantially exposed to similar harm. However, sex differences in the effects of LDCT screening among INS remain largely unexplored, and population-based studies are unable to effectively evaluate this aspect if LCS is implemented for both never and ever smokers. Therefore, we conducted a multicenter, individual-level cohort study focusing on Asian INS who underwent opportunistic LDCT screening to compare outcomes between women and men.

## Methods

### Study Design and Participants

We conducted a retrospective multicenter cohort study involving asymptomatic INS aged 50 to 80 years who underwent opportunistic LCS with LDCT between January 1, 2009, and December 31, 2021. This study was approved by the institutional review board of Seoul National University Bundang Hospital and Seoul National University Hospital. The requirement for informed consent was waived due to the minimal risk to participants. We followed the Strengthening the Reporting of Observational Studies in Epidemiology (STROBE) reporting guideline.

All participants voluntarily underwent LDCT screening, which was not covered by the national cancer screening program and national health insurance, and as part of health check-ups that provide LDCT according to each individual’s will and own payment. The study was conducted at 2 large, distinct, tertiary hospital-affiliated health checkup centers in different provinces of South Korea (Seoul National University Bundang Hospital, Gyeonggi-do, and Seoul National University Hospital, Seoul). (See eMethods in Supplement 1 for detailed information on the screening cohort and LDCT scanner protocols.) Individuals with screen-detected noncalcified nodules were referred to the pulmonary division to receive further follow-up or diagnostic evaluations according to guidelines at the time.^25,26^ For individuals with no abnormal findings at baseline LDCT, there were no specific recommendations to undergo additional screening rounds. Therefore, we determined that all the participants underwent a one-off LDCT screening, and all additional imaging received during follow-up after the negative baseline scan were determined post screening. During the study period, 43 045 individuals aged 50 to 80 years underwent LDCT screening (eFigure 1 in Supplement 1). After excluding individuals who had ever smoked, had an unknown smoking history, or had a previously diagnosed lung cancer, 21 062 participants (16 133 women and 4929 men) were eligible for final analyses.

### Variables and Outcome Measures

For this study, screening LDCT findings and radiologic characteristics of detected nodules were retrospectively classified using the Lung Reporting and Data System (Lung-RADS) criteria (version 2022).^27^ For those with multiple nodules, the nodule with the highest Lung-RADS category was considered the representative nodule. For participants with a present nodule on screening LDCT, medical records on the clinical course, including follow-up, invasive procedures for pathologic evaluation, and final diagnostic results such as lung cancer diagnosis (ie, screen-detected lung cancer, defined as lung cancer diagnosed from a nodule detected at baseline screening), were obtained. Participants with negative baseline LDCT screening findings were tracked using medical records through December 2022 to ascertain any subsequent diagnosis of lung cancer (ie, postscreen lung cancer, defined as lung cancer diagnosed by symptoms or other opportunistic chest imaging in addition to negative baseline screening). For patients diagnosed with lung cancer, initial screening results and subsequent imaging and diagnostic work-up were reviewed to determine whether it could be identified as screen-detected lung cancer. Final pathological reports, staging reports, and records of initial treatment and outcomes were also obtained. The pathological findings of lung cancer in this study were reviewed and classified according to the 2021 WHO Classification.^28^ Lung cancer staging was based on the eighth edition of the International Association for the Study of Lung Cancer and American Joint Committee on Cancer Stage Classification of non–small-cell lung cancer.^2,29^ Lung cancer stage was defined as the pathological stage if surgically resected; otherwise, the clinical stage was determined.

The primary outcomes of this study were lung cancer diagnosis and lung cancer–specific death (LCSD), compared between women and men. Moreover, associations between sex and main outcomes were evaluated with adjustments for predefined covariates. Vital statuses of all participants were determined by linking records from the Statistics Korea database, supplemented by the Korea Statistics Promotion Institute. The vital status, date of death, and cause of death were determined until December 31, 2022. LCSD was defined as death caused by lung cancer. Cause of death was further verified by the investigators using available medical records.

### Statistical Analysis

Descriptive analyses were performed using mean and SD for continuous variables and frequencies and proportions for categorical variables to describe the demographic, clinical, radiologic, and pathologic characteristics of the participants. Comparisons between women and men were conducted using Student*t* test for continuous variables and Pearson χ^2^ test or Fisher exact test for categorical variables. Cumulative incidence of lung cancer diagnosis during follow-up from baseline screening was evaluated using medical records, with cumulative incidence of LCSD using follow-up data censoring records linked from the Statistics Korea Database. Individuals who did not die during the study period were censored on December 31, 2022. Cox proportional hazards models adjusted for age and family history of lung cancer were used for estimations and comparisons. No covariates in the multivariable models had missing values. In addition, survival analyses were conducted, considering LCSD as the outcome of interest and death from causes other than lung cancer as a competing event. Sex differences in the cumulative incidence of LCSD were tested using the Gray model.^30,31^ For patients diagnosed with lung cancer, survival estimation from diagnosis was performed using Kaplan-Meier analysis with log-rank tests. Statistical analyses were performed using STATA version 16.0 (StataCorp). Hazard ratios (HRs), subhazard ratios (SHRs) and 95% CIs were calculated, and 2-sided *P* < .05 were considered statistically significant. Data were retrospectively analyzed from November 2023 to June 2024.

## Results

### Participant Characteristics and Screening Results

Baseline demographic characteristics and screening results of 21 062 participants (16 133 [76.6%] women and 4929 [23.4%] men) are described in Table 1. Overall, the mean (SD) participant age was 59.8 years, with no significant difference between the sexes. A total of 2423 participants, including 1891 women (11.7%) and 532 men (10.8%), had a family history of lung cancer. At baseline screening, 963 women (6.0%) and 328 men (6.7%) were detected with pulmonary nodules categorized as Lung-RADS 3 or higher. With baseline screening, 176 individuals (139 [0.9%] women and 37 [0.8%] men) were diagnosed with lung cancer (screen-detected). During a mean follow-up of 83.3 months from baseline screening, LCSD was reported in 8 women (<0.1%) and 3 men (<0.1%).

**Table 1.** Characteristics of Individuals With No Smoking History Who Received LDCT Screening

Characteristics	Participants, No. (%)		
	Total (n = 21 062)	Women (n = 16 133)	Men (n = 4929)
Age at baseline screening			
Mean (SD), y	59.8 (7.2)	59.8 (7.1)	59.9 (7.4)
50-59 y	11 573 (54.9)	8850 (54.9)	2723 (55.2)
60-69 y	6934 (32.9)	5379 (33.3)	1555 (31.5)
70-80 y	2555 (12.1)	1904 (11.8)	651 (13.2)
Family history of lung cancer	2423 (11.5)	1891 (11.7)	532 (10.8)
Individuals with detected lung nodule^a^	4309 (20.5)	3308 (20.5)	1001 (20.3)
Lung-RADS category at baseline LDCT screening			
1	16 753 (79.5)	12 825 (79.5)	3928 (79.7)
2	3018 (14.3)	2345 (14.5)	673 (13.7)
3	715 (3.4)	528 (3.3)	187 (3.8)
4A	392 (1.9)	313 (1.9)	79 (1.6)
4B or 4X	184 (0.9)	122 (0.8)	62 (1.3)
Follow-up duration, mean (SD), mo	83.8 (41.7)	83.0 (41.0)	86.6 (43.7)

Abbreviations: LDCT, low-dose chest computed tomography; Lung-RADS, Lung Imaging Reporting and Data System.

^a^ Screen-detected nodule categorized as Lung-RADS 2 or higher.

### Characteristics and Clinical Course of Positive Screening Results

The characteristics and sex differences among individuals with screen-detected nodules (at least Lung-RADS 2) are described in Table 2. Compared with nodules detected in women, nodules detected in men were more likely to be of the solid type (as opposed to subsolid) and larger in size. Lung cancer detection rates according to baseline Lung-RADS category 2 were 26 of 2345 (1.1%) in women and 6 of 673 (0.9%) in men; for Lung-RADS category 3, 32 of 528 (6.1%) for women and 9 of 187 (4.8%) for men; for Lung-RADS category 4A, 34 of 313 (10.9%) in women and 6 of 79 (7.6%) in men; and for Lung-RADS category 4B/4X, 47 of 122 (38.5%) in women and 16 of 62 (25.8%) in men. The modality for the initial diagnostic approach did not differ significantly between women and men, with video-assisted surgical biopsy being the main strategy (115 of 139 [82.7%] for women and 32 of 37 [86.5%] for men).

**Table 2.** Characteristics and Comparison Between Women and Men Who Never Smoked With Nodules Detected at Baseline LDCT Screening

Characteristics	Participants, No. (%)			*P* value
	Total (n = 4309)	Women (n = 3308)	Men (n = 1001)	
Age, mean (SD), y	60.7 (7.5)	60.7 (7.5)	60.6 (7.4)	.81
Location of dominant nodule				
Right upper lobe	1048 (24.3)	813 (24.6)	235 (23.5)	.79
Right middle lobe	559 (13.0)	428 (12.9)	131 (13.1)	
Right lower lobe	970 (22.5)	732 (22.1)	238 (23.8)	
Left upper lobe	922 (21.4)	706 (21.3)	216 (21.6)	
Left lower lobe	810 (18.8)	629 (19.0)	181 (18.1)	
Individuals with multiple nodules	1392 (32.3)	1044 (31.6)	348 (34.8)	.06
Nodule type				
Solid	2722 (63.2)	2053 (62.1)	669 (66.8)	.003
Part-solid	511 (11.9)	388 (11.7)	123 (12.3)	
Pure GGN	1076 (25.0)	867 (26.2)	209 (20.9)	
Nodule size at first detection, mean (SD), mm	6.6 (4.6)	6.5 (4.3)	7.0 (5.6)	.002
Lung-RADS category				
2	3018 (70.0)	2345 (70.9)	673 (67.2)	<.001
3	715 (16.6)	528 (16.0)	187 (18.7)	
4A	392 (9.1)	313 (9.5)	79 (7.9)	
4B or 4X	184 (4.3)	122 (3.7)	62 (6.2)	
Diagnosed as lung cancer	176 (4.1)	139 (4.2)	37 (3.7)	.47
Initial diagnostic evaluation for lung cancer, No./total No. (%)				
VATS biopsy	147/176 (83.5)	115/139 (82.7)	32/37 (86.5)	.51
Percutaneous needle biopsy	21/176 (11.9)	16/139 (11.5)	5/37(16.5)	
Bronchoscopic biopsy	4/176 (2.3)	4/139 (2.9)	0/37 (0)	
Radiotherapy without pathologic confirmation	4/176 (2.3)	4/139 (2.9)	0/37 (0)	

Abbreviations: GGN, ground-glass nodules; LDCT, low-dose chest computed tomography; Lung-RADS, Lung Imaging Reporting and Data System; VATS, video-assisted thoracic surgery.

### Characteristics and Treatment of Lung Cancers

Among the 176 screen-detected lung cancer cases, 141 (80.1%; 110 of 139 [79.1%] in women and 31 of 37 [83.8%] in men) were initially diagnosed from subsolid nodules, most of those being classified as part-solid (Table 3). The mean time from first nodule detection to lung cancer diagnosis was 26.3 months, with no significant difference between women and men. For both sexes, the predominant histological type of lung cancer was adenocarcinoma, accounting for 96.0% of all screen-detected lung cancer cases (133 of 139 women [95.7%] and 36 of 37 men [97.3%]). The proportion of lung cancer cases diagnosed at a localized stage (stage 0–II) was 133 of 139 women (95.7%) and 35 of 37 men (94.6%). Of the 168 stage 0 to II cancers, 163 (97.0%) were treated with surgery, and 5 (3.0%) were treated with stereotactic body radiation therapy. Among all patients with screen-detected lung cancer, 167 of 176 (94.9%) received surgical resection for initial treatment.

**Table 3.** Characteristics and Sex Differences of Screen-Detected Lung Cancer Cases

Characteristics	Participants, No. (%)			*P* value
	Total (n = 176)	Women (n = 139)	Men (n = 37)	
Age at diagnosis, mean (SD), y	65.0 (8.0)	64.7 (8.1)	66.1 (7.7)	.31
Nodule type at first detection				
Solid	35 (19.9)	29 (20.9)	6 (16.2)	.64
Part-solid	112 (63.6)	86 (61.9)	26 (70.3)	
Pure GGN	29 (16.5)	24 (17.3)	5 (13.5)	
Time from baseline screening to lung cancer diagnosis, mean (SD), mo	26.3 (37.6)	27.5 (37.5)	21.7 (38.4)	.41
Cancer histology and predominant subtype				
Adenocarcinoma	169 (96.0)	133 (95.7)	36 (97.3)	.15
AIS	5 (2.8)	5 (3.6)	0 (0)	
MIA	40 (22.7)	30 (21.6)	10 (27.0)	
Lepidic predominant	18 (10.2)	15 (10.8)	3 (8.1)	
Acinar predominant	54 (30.7)	44 (31.7)	10 (27.0)	
Papillary predominant	20 (11.4)	17 (12.2)	3 (8.1)	
Microinvasive papillary predominant	3 (1.7)	1 (0.7)	2 (5.4)	
Solid predominant	4 (2.3)	4 (2.9)	0	
Invasive mucinous adenocarcinoma	8 (4.5)	7 (5.0)	1 (2.7)	
Mixed or unspecified	17 (9.7)	10 (7.2)	7 (18.9)	
Adenosquamous carcinoma	1 (0.6)	1 (0.7)	0	
Squamous cell carcinoma	1 (0.6)	0	1 (2.7)	
Other non–small-cell carcinoma^a^	5 (2.8)	5 (3.6)	0	
Lung cancer stage				
0 (AIS)	5 (2.8)	5 (3.6)	0	.28
IA	142 (80.7)	113 (81.3)	29 (78.4)	
IB	17 (9.7)	13 (9.4)	4 (10.8)	
IIA	1 (0.6)	0	1 (2.7)	
IIB	3 (1.7)	2 (1.4)	1 (2.7)	
IIIA	4 (2.3)	2 (1.4)	2 (5.4)	
IIIB	1 (0.6)	1 (0.7)	0	
IIIC	0	0	0	
IV	3 (1.7)	3 (2.2)	0	
Initial treatment				
Surgery	167 (94.9)	131 (94.2)	36 (97.3)	.12
Radiotherapy	5 (2.8)	5 (3.6)	0	
CCRT	1 (0.6)	0	1 (2.7)	
Chemotherapy	3 (1.7)	3 (2.2)	0	
Total time of follow-up from diagnosis, mean (SD), mo	64.0 (41.7)	62.0 (41.7)	71.4 (41.7)	.22
Lung cancer–specific death	4 (2.3)	4 (2.9)	0	.29
Death from other causes	7 (4.0)	5 (3.6)	2 (5.4)	.61

Abbreviations: AIS, adenocarcinoma in situ; CCRT, concurrent chemoradiation therapy; GGN, ground-glass nodules; MIA, minimally invasive adenocarcinoma.

^a^ Includes poorly differentiated non–small-cell carcinomas and cases that underwent direct radiotherapy without pathological confirmation.

eTable 1 in Supplement 1 describes the evaluation of the total 196 lung cancer cases diagnosed in this cohort, including 20 postscreen lung cancer cases (detected by symptoms or other opportunistic chest imaging after a negative baseline screening). The proportions of histological subtypes, stage distribution, and choice of initial treatment did not differ significantly between sexes.

### Sex Differences in Lung Cancer Risk and Related Outcomes

Among 21 062 participants who underwent LCS, during the follow-up period of 83.3 months from baseline LDCT, there were 286 deaths (1.8%) with 8 LCSD among women and 154 deaths (3.1%) with 3 LCSD among men, according to the matched Statistics Korea Database. One LCSD case in a female participant was reported after 127.4 months following a negative screening LDCT, where medical records could not censor the diagnostic characteristics of postscreen lung cancer due to management outside the health care facilities in which this study was conducted. Therefore, this case was not included in the analyses for lung cancer diagnosis; however, it was included as an LCSD case in the mortality analyses for all participants.

**Figure 1.**
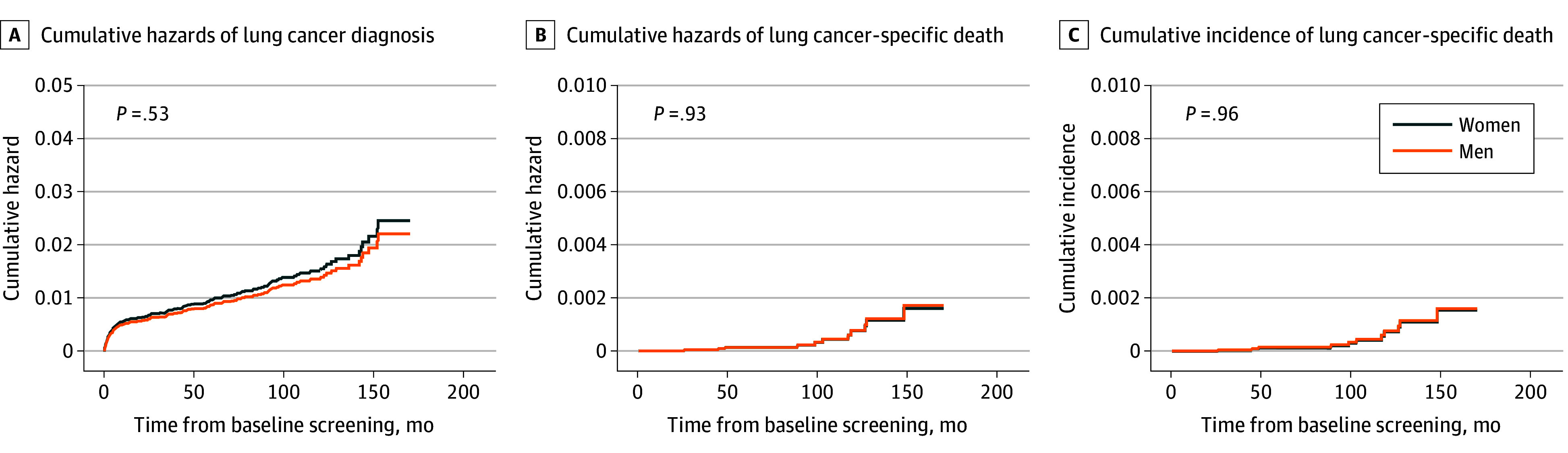
Cumulative Hazards of Lung Cancer Diagnosis and Lung Cancer–Specific Death and Cumulative Incidence of Lung Cancer–Specific Death for Women vs Men The curves in this figure compare women and men for cumulative hazards of lung cancer diagnosis (A), cumulative hazards of lung cancer–specific death (B), and cumulative incidence of lung cancer–specific death by competing risk regression considering death from causes other than lung cancer as a competing event (C). Adjusted for age and family history of lung cancer.

**Figure 2.**
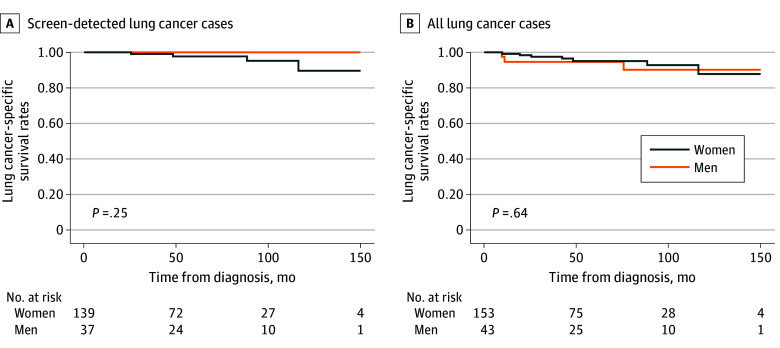
Lung Cancer–Specific Survival Curves, Stratified by Sex Lung cancer–specific survival curves of screen-detected lung cancer cases (A) and all lung cancer cases (B), stratified by sex.

Among all participants who underwent LCS, after adjusting for age and family history of lung cancer, no significant associations were observed between sex and the cumulative hazards of lung cancer diagnosis (adjusted hazard ratio HR [aHR], 0.90 [95% CI, 0.64-1.26] for men vs women) (Figure 1A; eTable 2 in Supplement 1) or the cumulative hazards of LCSD (aHR, 1.06 [95% CI, 0.28-4.00] for men vs women) (Figure 1B; eTable 3 in Supplement 1). Multivariable competing risk regression with adjustments for age and family history revealed similar results, with no significant association between sex and the cumulative incidence of LCSD (aHR, 1.03 [95% CI, 0.27-3.96] for men vs women) (Figure 1C; eTable 4 in Supplement 1). For both the 176 screen-detected and 196 all-diagnosed lung cancer cases (including postscreen cancers), there were no significant differences in lung cancer–specific survival between women and men (Figure 2A and 2B). The estimated 5-year lung cancer–specific survival rates were 97.7% and 100% for women and men, respectively, for screen-detected lung cancer cases; and 95.2% and 94.7% for women and men, respectively, for all lung cancer cases.

## Discussion

In this multicenter cohort study of individuals with no smoking history who underwent opportunistic LDCT screening, we examined differences in screening results, lung cancer diagnoses, and related prognostic outcomes between women and men. Our pragmatic design, which included asymptomatic individuals who voluntarily underwent LDCT screening, provides insights into the actual status of LCS among INS in East Asia. Notably, with baseline screening, the detection rate of lung cancer in men was 0.8%, which was similar to women (0.9%) and comparable with baseline screening detection rates reported from the NLST (1.1%) and NELSON trial (0.9%), which screened populations with substantial cigarette smoke exposure.^5,6^ A large proportion of screen-detected lung cancers identified in men were detected as subsolid nodules (83.8%), exhibited adenocarcinoma as the predominant histological type (97.3%), and were diagnosed at an early stage, leading to a high surgical resection rate (97.3%) and excellent survival outcomes (5-year lung cancer–specific survival rate of 100%). Screening parameters and prognostic outcomes were similar with those observed in women. Overall, there were no significant sex differences in the risk of lung cancer detection or LCSD. This study, to our knowledge, provides novel individual-level data on sex differences in LDCT screening outcomes among INS in East Asia, where opportunistic LCS programs that include nonsmokers are popular.^16,17,19^

Sex differences in lung cancer have been identified in various aspects including epidemiology and survival outcomes.^32,33^ In the context of screening, large trials assessing the efficacy of LDCT screening in heavy smokers have suggested that LCS may be more beneficial for women than for men. In the extended analysis of the National Lung Screening Trial (NLST), risk ratios (RRs) for LCSD in dilution-adjusted analysis were 0.80 and 0.95 in women and men, respectively.^34^ In the NELSON trial, the magnitude of LCSD reduction was higher in women (33%) than in men (24%).^6^ Regarding LCS in INS, few studies have reported the efficacy of LDCT-based screening. Recognizing distinct epidemiological patterns, most investigations have been conducted in East Asia, where the proportion of lung cancer incidence and related mortality in INS is relatively high and increasing.^35,36^ However, sex differences in the screening outcomes of this population have been rarely evaluated. Cohort studies from Japan, South Korea, and Taiwan demonstrated a notable lung cancer prevalence of 0.5% to 2.6% among screened INS, with the majority of cancers diagnosed at an early stage.^15,18,37,38^ Although supporting the possibility of effectively performing LCS for INS, these findings necessitate further evaluation for generalizability.^12^ Moreover, identifying sex differences is important, considering the distinct epidemiological patterns observed in LCINS.

Epidemiological studies from Asia have revealed a higher incidence of LCINS in men than women.^39,40^ In a population-based study from Korea, the rate ratio of LCINS was 2.4 for men vs women.^40^ Similarly, a Chinese cohort study identified male sex as an independent risk factor for LCINS (HR = 0.67 for women vs men), therefore including sex in the lung cancer prediction model for INS.^41^ However, results from Asian LCS studies on INS have revealed mixed results, ranging from no significant sex difference to a higher incidence of lung cancer in women than in men.^18,42^ In a recent meta-analysis by Triphuridet et al,^43^ the RR of lung cancer diagnosed in a screening cohort was 1.78 for women who never smoked vs men who never smoked. This discrepancy between epidemiological findings and LCS results is likely attributed to the substantial proportion of younger women (aged <50 years) in screening studies.^43^ A prior study from our group revealed that among 4545 INS with screen-detected subsolid nodules, 36.5% were aged less than 50 years and 67.7% were women.^44^ Pre- and minimally invasive adenocarcinomas diagnosed from subsolid nodules would have contributed to the relatively higher incidence of lung cancer in the screening cohorts, including younger women who never smoked. The current study, which evaluated LCS results among INS aged 50–80 years, revealed similar rates of nodule detection and lung cancer diagnosis in women and men. The overall proportion of a positive screen, incidence and characteristics of lung cancer, with a predominance of adenocarcinomas, shown in our study align with findings from prior LCS studies on Asian population without smoking history of a similar average age, supporting the validity of our findings.^37,45,46^

Our findings have implications for the recently raised concerns about potential overdiagnosis with the widespread implementation of LCS in INS. Data from the Taiwan Lung Cancer Screening for Never Smoker Trial (TALENT) showed that 96.5% (307/318) of screen-detected lung cancers were stage 0–1 disease, mainly adenocarcinomas, and 19.2% (61/318) were diagnosed at stage 0 (ie, adenocarcinoma in situ).^38^ It is probable that a substantial proportion of these early-stage adenocarcinomas are not destined to cause mortality (ie, overdiagnosed lung cancers). Additional evidence of overdiagnosis in screening women who never smoked was recently provided by population-based ecological studies in Taiwan and China.^16,17^ Using data from the Taiwan National Cancer Registry, Gao et al^16^ reported a 6-fold increase in the incidence of stage 0 to I lung cancer in women between 2004 and 2018 following the large introduction of opportunistic LDCT screening in the mid-2000s. However, this increase was not accompanied by a decrease in the incidence of late-stage disease or LCSD.^16^ Similar results were reported by Wang et al^17^ from the Shanghai Cancer Registry, where the incidence of stage I cancer increased approximately 19-fold in women between 2002 and 2017 without a significant decline in stage II to IV disease. The upward trend in incidence was mainly observed in adenocarcinomas.^17^ Both studies from Taiwan and China revealed signals of lung cancer overdiagnosis in women who never smoked attributed to the low (<7%) smoking prevalence among women in these regions. However, population-based studies were unable to investigate the effect of opportunistic LDCT screening in men who never smoked given the higher smoking prevalence in men (>35%). Therefore, there is a lack of evidence regarding whether overdiagnosis in screened INS is particularly concentrated in women.^24^ In our study, which evaluated individual-level outcomes from an opportunistic LDCT screening cohort of INS with a long-term follow-up period (mean 83.8 months), the risk of LCSD was similar between the sexes. In addition, the proportion of screen-detected stage 0 to I disease (>89%), predominance of adenocarcinoma (>95%), and 5-year lung cancer–specific survival rates (>97%) were similar between women and men. Particularly, the 5-year survival rates were substantially higher than those reported in studies screening high-risk populations.^5,6^ Overall, our findings suggest that women who never smoked do not experience greater susceptibility to lung cancer overdiagnosis than men who never smoked when equally exposed to indiscriminate screening. This study, to our knowledge, is the first to document that the potential risk of overdiagnosis would be equal between women and men when LCS is implemented routinely for INS.

### Limitations

Our study has limitations. First, due to the retrospective nature of the study, the strategies for LDCT screening, follow-up, and nodule management were not strictly controlled. Second, our results were derived from a health center-based design; therefore, the participants may not fully reflect the general population. To minimize selection bias, we included asymptomatic individuals who voluntarily underwent LCS at 2 large health checkup centers located in different provinces of South Korea. Third, there is a possibility of underestimation of lung cancer incidence because some participants may have sought management outside the health care facilities where this study was conducted. However, this would be a rare event having low clinical impact because all LCSDs were captured by the link to a nationwide mortality statistics database, and diagnostic characteristics were uncensored by hospital-based medical records in only one case. Additionally, other than family history, data on exposures to possible risk factors for LCINS, such as air pollution, occupational factors, and second-hand smoke, were unavailable.

## Conclusions

In this cohort study of individuals without smoking history who underwent LDCT screening, men and women showed similar outcomes in terms of nodule detection rate, lung cancer diagnosis, stage distribution, and LCSD. Our findings suggest that when routinely screened, men who never smoked would experience similar susceptibility to the risk of overdiagnosis as women who never smoked with the little to no benefit and the potential for harm in this group. This highlights the fact that routine screening of INS must be approached with caution at this point, regardless of the sex. Future studies are warranted to investigate the risk factors and prediction models for the selection of suitable candidates of both sexes with no smoking history who would truly benefit from LDCT screening. In particular, randomized clinical trials including INS must be considered to obtain relevant evidence.

## Notes

Supplement 1. **eMethods.** Supplementary Materials **eFigure.** Flow Diagram of the Study Population **eTable 1.** Characteristics and Sex Differences of All Lung Cancer Cases **eTable 2.** Univariable and Multivariable Cox-Proportional Regression Analysis for Lung Cancer Diagnosis **eTable 3.** Univariable and Multivariable Cox-Proportional Regression Analysis for Lung Cancer-Specific Death **eTable 4.** Univariable and Multivariable Competing Risk Regression Analysis for Lung Cancer–Specific Death

Supplement 2.
Data Sharing Statement
