# Supplementary material for: Gender Disparities and Lung Cancer Screening Outcomes Among Individuals Who Have Never Smoked
Source: JAMA Netw Open. 2025 Jan 15;8(1):e2454057. doi: 10.1001/jamanetworkopen.2024.54057 (PMC11736501; doi:10.1001/jamanetworkopen.2024.54057)
Supplement: Supplement 1. — eMethods. Supplementary Materials eFigure. Flow Diagram of the Study Population eTable 1. Characteristics and Sex Differences of All Lung Cancer Cases eTable 2. Univariable and Multivariable Cox-Proportional Regression Analysis for Lung Cancer Diagnosis eTable 3. Univariable and Multivariable Cox-Proportional Regression Analysis for Lung Cancer-Specific Death eTable 4. Univariable and Multivariable Competing Risk Regression Analysis for Lung Cancer–Specific Death [file jamanetwopen-e2454057-s001.pdf]

Supplemental Online Content

Kim YW, Joo D, Kim SY, et al. Gender disparities and lung cancer screening outcomes among individuals who have never smoked. *JAMA Netw Open.* 2025;8(1):e2454057. doi:10.1001/jamanetworkopen.2024.54057

eMethods. Supplementary Materials

eFigure. Flow Diagram of the Study Population

eTable 1. Characteristics and Sex Differences of All Lung Cancer Cases

eTable 2. Univariable and Multivariable Cox-Proportional Regression Analysis for Lung Cancer Diagnosis

eTable 3. Univariable and Multivariable Cox-Proportional Regression Analysis for Lung Cancer-Specific Death

eTable 4. Univariable and Multivariable Competing Risk Regression Analysis for Lung Cancer–Specific Death

This supplemental material has been provided by the authors to give readers additional information about their work.

© 2025 Kim YW et al. *JAMA Network Open*.

eMethods. Supplementary Methods

Study participants

The study cohort comprised asymptomatic individuals without smoking history and aged 50–80 years who underwent opportunistic LCS with LDCT between January 1, 2009, and December 31, 2021 two large, distinct, tertiary hospital-affiliated health checkup centers in different provinces of South Korea. The role of the health checkup centers mainly includes health check-up and screening, and differ from medical clinics that manage patients with symptoms and underlying lung disease. Among those who underwent LDCT screening, self-administered questionnaires were administered with screening to collect information on the smoking status and intensity and family history of lung cancer. INS were defined as individuals who had smoked <100 cigarettes or had never smoked during their lifetime. Participants with a previous diagnosis of lung cancer were excluded. Individuals with screen-detected nodules were referred to the pulmonary division to receive further follow-up or diagnostic evaluations according to guidelines at the time. Based on the guidelines, decision to perform an invasive biopsy mainly depended on the radiologic characteristics and clinical course of the nodule and was not additionally weighted by other demographic factors.

LDCT protocol and scanner information

Screening LDCT scans were conducted at a peak tube voltage of 100 or 120 kV and a reference tube current of 20–70 mA using one of the following multidetector row scanners: Mx-8000 IDT 1, Mx-8000 IDT 2, Mx-8000 IDT 16 (Philips Medical Systems, Cleveland, OH, USA), Brilliance-64, Brilliance iCT 256 (Philips Medical Systems, Best, The Netherlands), Sensation 16, SOMATOM Definition, or SOMATOM Definition Force (Siemens Medical Solutions, Forchheim, Germany). All CT images were reconstructed with ≤3-mm slices in the axial plane

© 2025 Kim YW et al. *JAMA Network Open*.

and 3-mm slices in the coronal plane, and were initially stored in a dedicated electronic Picture Archiving and Communication System (PACS). All CT scans were initially reviewed and reported by board-certified chest radiologists, defining non-calcified nodules ≥4 mm as positive pulmonary nodules. Two pulmonologists (YWK and DHJ) additionally reviewed all the LDCT scans and classified the radiologic findings according to the Lung-RADS version 2022.

© 2025 Kim YW et al. *JAMA Network Open*.

eFigure 1. Flow diagram of the study population

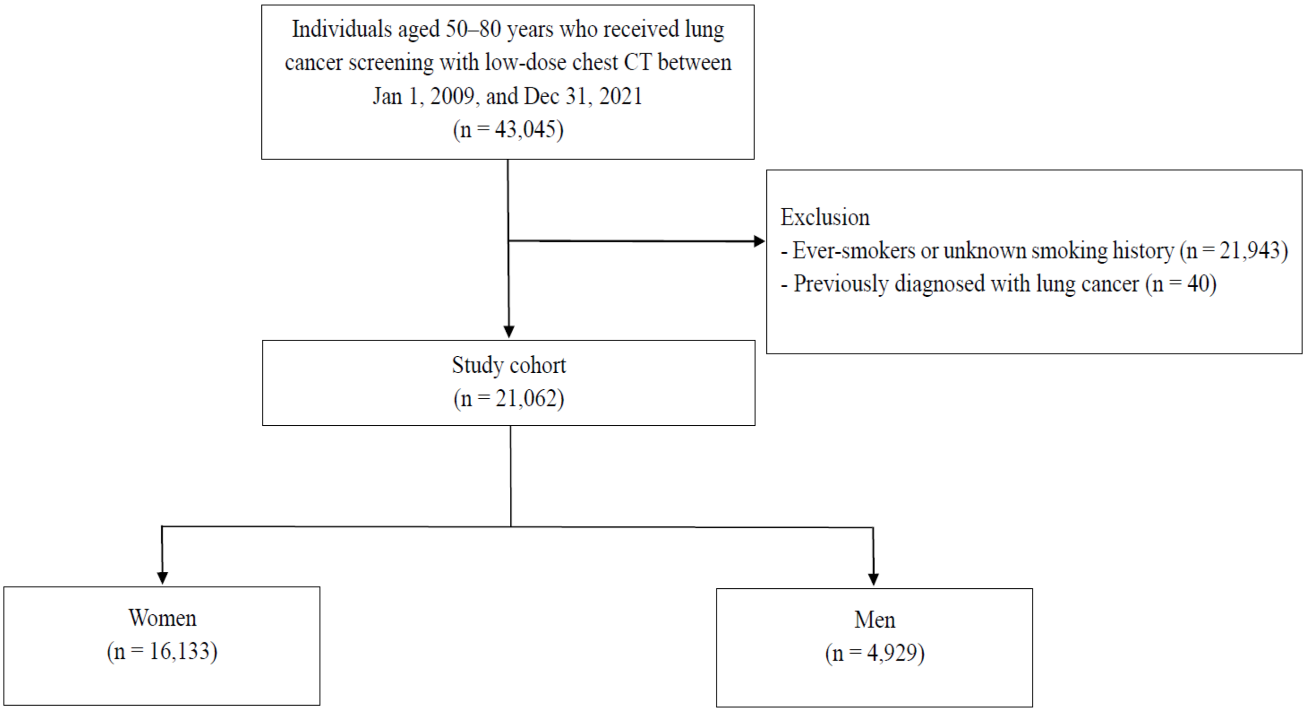

© 2025 Kim YW et al. *JAMA Network Open*.

eTable 1. Characteristics and sex differences of all lung cancer cases

	Total (n = 196)	Women (n = 153)	Men (n = 43)	*P* value
Age at diagnosis, mean (SD)	65.2 (7.9)	65.0 (8.0)	66.1 (7.3)	0.39
Nodule type at first detection				0.92
Solid	46 (23.5)	36 (23.5)	10 (23.3)	
Part-solid	119 (60.7)	92 (60.1)	27 (62.8)	
Pure GGN	31 (15.8)	25 (16.3)	6 (14.0)	
Time from first screening to lung cancer diagnosis, months, mean (SD)	31.1 (40.3)	31.7 (38.9)	31.7 (45.5)	0.99
Cancer histology and predominant subtype, n (%)				0.03
Adenocarcinoma	187 (95.4)	146 (95.4)	41 (95.3)	
AIS	6 (3.1)	6 (3.9)	0 (0)	
MIA	41 (20.9)	31 (20.3)	10 (23.3)	
Lepidic predominant	19 (9.7)	15 (9.8)	4 (9.3)	
Acinar predominant	59 (30.1)	48 (31.4)	11 (25.6)	
Papillary predominant	22 (11.2)	18 (11.8)	4 (9.3)	
Microinvasive papillary predominant	3 (1.5)	1 (0.7)	2 (4.7)	
Solid predominant	4 (2.0)	4 (2.6)	0 (0)	
Invasive mucinous adenocarcinoma	9 (4.6)	8 (5.2)	1 (2.3)	
Mixed or unspecified	24 (12.2)	15 (9.8)	9 (20.9)	
Adenosquamous carcinoma	1 (0.5)	1 (0.7)	0 (0)	
Squamous cell carcinoma	2 (1.0)	0 (0)	2 (4.7)	
Other non-small cell carcinoma*	6 (3.1)	6 (3.9)	0 (0)	
Lung cancer stage, n (%)				0.78
0	6 (3.1)	6 (3.9)	0 (0)	
IA	150 (76.5)	118 (77.1)	32 (74.4)	
IB	18 (9.2)	14 (9.2)	4 (9.3)	
IIA	2 (1.0)	1 (0.7)	1 (2.3)	
IIB	3 (1.5)	2 (1.3)	1 (2.3)	
IIIA	5 (2.6)	3 (2.0)	2 (4.7)	

© 2025 Kim YW et al. *JAMA Network Open*.

IIIB	1 (0.5)	1 (0.7)	0 (0)	
IIIC	1 (0.5)	1 (0.7)	0 (0)	
IV	10 (5.1)	7 (4.6)	3 (7.0)	
Initial treatment, n (%)				0.43
Surgery	179 (91.3)	140 (91.5)	39 (90.7)	
Radiotherapy	5 (2.6)	5 (3.3)	0 (0)	
CCRT	2 (1.0)	1 (0.7)	1 (2.3)	
Chemotherapy	10 (5.1)	7 (4.6)	3 (7.0)	
Total months of follow from diagnosis, mean (SD)	60.4 (41.9)	59.2 (41.7)	65.0 (42.9)	0.43
Lung cancer-specific death, n (%)	10 (5.1)	7 (4.6)	3 (7.0)	0.52
Death from other causes, n (%)	8 (4.1)	6 (3.9)	2 (4.7)	0.83

NA: not applicable, CCRT: concurrent chemoradiation therapy, SD: standard deviation, GGN: ground-glass nodules, AIS: adenocarcinoma *in situ*, MIA: minimally invasive adenocarcinoma

* Includes poorly differentiated non-small cell carcinomas and cases that underwent direct radiotherapy without pathological confirmation.

© 2025 Kim YW et al. *JAMA Network Open*.

eTable 2. Univariable and multivariable Cox-proportional regression analysis for lung cancer diagnosis

Variable	Univariate		Multivariate	
	HR (95% CI)	p value	HR (95% CI)	*P* value
Sex (men)	0.91 (0.65–1.27)	0.58	0.90 (0.64–1.26)	0.53
Age at baseline screening	1.05 (1.03–1.07)	<0.001	1.05 (1.03–1.07)	<0.001
Family history of lung cancer	1.88 (1.33–2.66)	<0.001	1.99 (1.40–2.81)	<0.001

HR: hazard ratio, CI: confidence interval

© 2025 Kim YW et al. *JAMA Network Open*.

eTable 3. Univariable and multivariable Cox-proportional regression analysis for lung cancer-specific death

Variable	Univariate		Multivariate	
	**HR (95% CI)**	**p value**	**HR (95% CI)**	***P* value**
Sex (male)	1.05 (0.28–3.96)	0.95	1.06 (0.28–4.00)	0.93
Age at baseline screening	1.10 (1.02–1.19)	0.02	1.10 (1.02–1.19)	0.01
Family history of lung cancer	1.85 (0.40–8.56)	0.43	2.13 (0.46–9.92)	0.33

HR: hazard ratio, CI: confidence interval

© 2025 Kim YW et al. *JAMA Network Open*.

eTable 4. Univariable and multivariable competing risk regression analysis for lung cancer–specific death

Variable	Univariate		Multivariate	
	**SHR (95% CI)**	**p value**	**HR (95% CI)**	***P* value**
Sex (male)	1.04 (0.27–3.94)	0.96	1.03 (0.27–3.96)	0.96
Age at baseline screening	1.09 (1.02–1.17)	0.008	1.10 (1.03–1.17)	0.006
Family history of lung cancer	1.86 (0.40–8.74)	0.43	2.13 (0.46–9.80)	0.33

SHR: subhazard ratio, CI: confidence interval

© 2025 Kim YW et al. *JAMA Network Open*.
