# Supplementary material for: Gender Disparities and Lung Cancer Screening Outcomes Among Individuals Who Have Never Smoked
Source: JAMA Netw Open. 2025 Jan 15;8(1):e2454057. doi: 10.1001/jamanetworkopen.2024.54057 (PMC11736501; doi:10.1001/jamanetworkopen.2024.54057)
Supplement: Supplement 2. — Data Sharing Statement [file jamanetwopen-e2454057-s002.pdf]

Data Sharing Statement

Kim. Gender Disparities and Lung Cancer Screening Outcomes Among Individuals Who Have Never Smoked. *JAMA Netw Open*. Published January 15, 2025. doi:10.1001/jamanetworkopen.2024.54057

Data
Data available: Yes
Data types: Deidentified participant data
How to access data: Available from the corresponding author upon reasonable and ethically approved request
When available: With publication

Supporting Documents
Document types: None

Additional Information
Who can access the data: Researchers whose proposed use of the data has been approved
Types of analyses: Meta-analysis
Mechanisms of data availability: After approval
